# Effects of Remotely Supervised Physical Activity on Health Profile in Frail Older Adults: A Randomized Controlled Trial Protocol

**DOI:** 10.3389/fnagi.2022.807082

**Published:** 2022-03-31

**Authors:** Xin Zhang, Jinwei Li, Xin Sui, Linqi Xu, Lanyu Zhu, Yue Pang, Tianzhuo Yu, Xiaoqian Lian, Tianyue Yu, Yuewei Li, Haiyan Xu, Feng Li

**Affiliations:** School of Nursing, Jilin University, Changchun, China

**Keywords:** remotely supervised, physical activity, intelligent system, frailty, health profile

## Abstract

**Background:**

Frailty is considered a major public health challenge of the 21st century, characterized by the decline of multiform body functions. Physical activity may be the most effective intervention to delay frailty. This study aims to verify the effect of remotely supervised physical activity on health profile in community-dwelling frail older adults.

**Design:**

This is a multicenter, three-blind, two-arm, and cohort randomized controlled study.

**Methods:**

The intelligent exercise rehabilitation management system (IERMS) is an integrated digital platform that involves evaluation, guidance, monitoring, and feedback. A total of 120 participants aged ≥ 65 years and diagnosed as frailty on the FRAIL scale will be recruited and randomly divided into two groups. Group 1 will receive a 12-week IERMS-based intervention, and Group 2 will receive the usual care. Data will be collected at baseline, 12 and 24 weeks. The primary outcome is the physical function, and secondary outcomes include gait parameters, psychology, and cognition measurements. Analyses will be performed using DSS statistics, version 25. *P* < 0.05 will be considered statistically significant.

**Conclusion:**

We believe that intervention plays a positive role in delaying the frailty. If our program is effective, we will provide a viable means to promote healthy aging in primary healthcare.

**Trial registration number:**

ChiCTR2100052286; Pre-results.

## Introduction

Frailty is a kind of clinical syndrome in older adults who are easily affected by stress. Physical frailty, originally defined by Fried et al. (2001), includes slow gait speed, weakness, self-reported exhaustion, low activity, and weight loss. Due to differences in regions, diagnostic criteria, and other factors, the prevalence of frailty is changed in different parts of the world; previous studies have shown that the prevalence ranged from 4 to 59% and increased with age (Choi et al., 2015; Hoogendijk et al., 2019). It is associated with the development of most chronic diseases, falls, fractures, disabilities, and other adverse outcomes (Theou et al., 2017; Hanlon et al., 2018). Fortunately, frailty is a dynamic reversible process, and measures can be taken to prevent it in advance (Lang et al., 2009).

The health benefits of physical activity have been widely recognized (Cheng et al., 2021). Proper physical activity can improve muscle, heart, and lung function and reduce the risk of high blood pressure, coronary heart disease, stroke, diabetes, cancer, depression, sleep disorders, falls, and fracture (de Labra et al., 2015; Dipietro et al., 2019; Mugueta-Aguinaga and Garcia-Zapirain, 2019). The WHO recommends that adults over 65 years of age engage in at least 150 min of moderate-intensity physical activity per week or at least 75 min of vigorous-intensity physical activity per week, or a combination of moderate and vigorous-intensity physical activity to achieve this amount of physical activity, with at least 10 min of continuous activity each time (van der Ploeg and Bull, 2020).

Recent research has found that physical activity may be the most effective intervention for frailty (Cheng et al., 2021). It is much more accomplished and effective when performed under supervision (Bonnefoy et al., 2012). Home-based supervised training has a better effect on strength and physical function and is more intense (Lacroix et al., 2017; Suikkanen et al., 2021). However, the allocation of health technicians is far from meeting the requirements of the training supervised by physiotherapists at home, so the current focus of primary healthcare is how to maximize the use of existing medical resources and benefit more people. Remote supervision based on wearable devices gradually attracts the attention of researchers (Garcia-Moreno et al., 2020; Zacharaki et al., 2020). This study aims to verify the effect of remotely supervised physical activity on health profile and observe the lingering effect in community-dwelling frail older adults.

## Methods

This study was designed and will be conducted and reported in keeping with the Consolidation Standards of Reporting (CONSORT) 2010 statement (Eldridge et al., 2016).

### Platform Delivery

The intelligent exercise rehabilitation management system (IERMS) was designed and developed by the research group independently (Xu et al., 2020). It is an integrating evaluation, guidance, monitoring, and feedback of an integrated intelligent motion rehabilitation management system. It consists of three parts: sensing device layer, management data layer, and application layer. The sensing layer collects the health data, including smart insoles, bracelets, and other common terminals; the management layer conducts data processing; and the application layer visualizes the health results, including websites, applications, and applets. Smart insole is a kind of sensing device that contains eight inertial and thin-film pressure sensors (FSR 400) and grants China an invention patent (Publication Patent Number: 201810114305.3).

### Study Design

**FIGURE 1:**
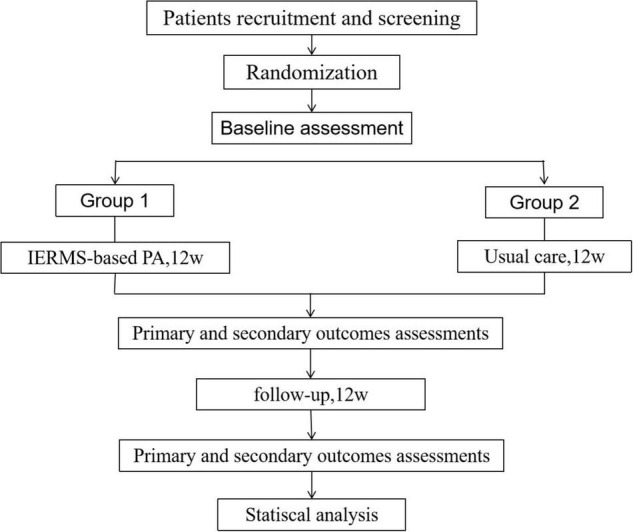
Flow chart of the experimental procedure.

A three-blind, two-arm, cohort randomized controlled trial will be conducted to evaluate the effects of IERMS-based physical activity on health performance in frail older adults (Registration number: ChiCTR2100052286)^1^. A total of 120 participants will be recruited according to the screening criteria from six community health service centers in Changchun, China, and the participants will be randomly divided into two groups. Participants in the first group will receive a 12-week IERMS-based intervention, and participants in the second group will receive a 12-week conventional care. Then, there will be a 12-week follow-up. Data will be collected at baseline, 12 and 24 weeks. The study design is shown in Figure 1. After the intervention, the same guidance will be given to the second group to ensure that more older adults benefit.

### Participants

#### Ethics Approval

The research protocol was approved by the Human Research Ethics Committee of the School of Nursing, Jilin University (HREC 2020122001). All participants will provide a signed informed consent when entering the study.

#### Eligibility and Recruitment

Participants will be recruited from six centers at the same time. Once there are 20 samples in a certain community, they will undergo the next step. Inclusion criteria are ≥ 65 years old, meeting the FRAIL scale for frail status, and familiar with the use of smartphones and tablets or computers. Exclusion criteria are nerve dysfunction (stroke, Parkinson’s disease, or lower limbs) with paraplegia, serious cardiovascular disease, cognitive impairment, continuous joint pain, severe muscle bone damage, life expectancy < 6 months of serious illness, serious hearing or visual impairment, severe depression or anxiety, other major diseases affecting training safety, hospitalization, and involvement in other clinical studies during this study.

#### Calculation of Sample Size

Based on *a priori* power analysis (G*Power 3.1.9.3) using a power of 0.90 and error probability of 0.05, a sample size of 50 participants will be required for each group to detect an assumed 20% difference in walking speed between the two groups. In addition, with an assumption of 15% dropout rate, a sample size of 120 participants will be initially targeted.

#### Randomization and Blinding

From each center, 20 participants will be recruited and stratified by gender and age; then, they will be randomly assigned to the first or second group in a 1:1 ratio through a computer-generated randomized list. This task will be performed independently by individuals not involved in the research process. All researchers will be divided into two teams: one team will be responsible for the guidance of group 1, and the other team conduct routine education, they will be ignorant of each other’s content. Throughout the intervention, neither the participants nor the researchers conducting the intervention or the data collection will be aware of the grouping. The study designer and the staff responsible for allocation concealment and data processing will be not permitted to participate in the whole intervention.

### Intervention

#### Training Based on Intelligent Exercise Rehabilitation Management System

**FIGURE 2:**
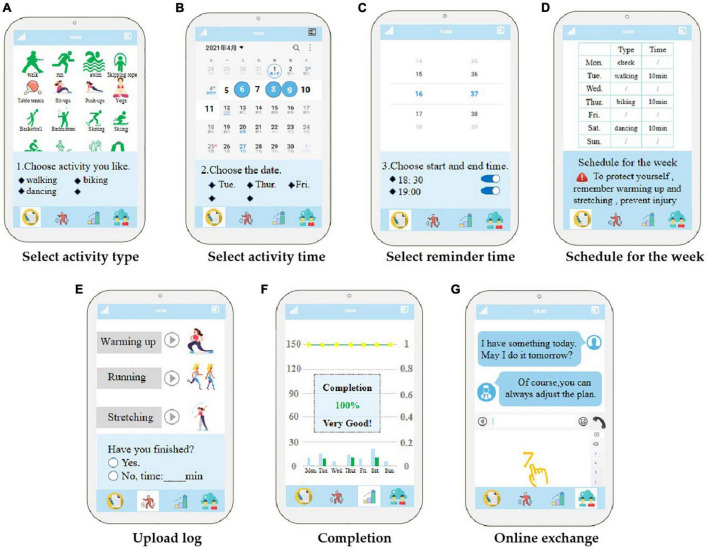
Prototype of the app user interface.

At baseline, all participants will undergo physical function, physical activity, psychology, and cognition assessments. These assessments will be repeated at 12 and 24 weeks. Participants in the first group will receive a pair of smart insoles and a patient-side APP installed on their phone and will learn all the functions of the system. According to the participants’ physical activity level at baseline, professional rehabilitation therapists will select the activity plan from the default scheme (Table 1); upload it to the cloud, which is the management data layer; and send the plan for the next week according to the participants’ weekly completion. If the activity goal of the week is not reached, the plan of the week will be continued. Once the current week’s activities are completed, the plan for the next phase will be carried out. Participants will choose the type of physical activity (Table 2) in the APP according to their preferences and choose the activity day of the week and the training time of each activity according to the target activity days. The system will automatically generate the particular week’s schedule according to participants’ choices and remind them at the time set by participants. At the end of each activity, participants will upload the daily completion of the activity and fill in the specific activity time for unfinished projects. They will have access to view the week’s activity plan at any time and generate the week’s activity schedule every Monday. During the workout, participants will be asked to wear smart insoles that monitor dynamic changes in gait parameters and synchronize them to the cloud in real time. In addition, patients will communicate with professionals through a short messaging service built into the APP and receive feedback within 24 h. The APP interface is shown in Figure 2. Before the start of each training, the user will receive a reminder as follows:

**TABLE 1:** Weekly training schedule.

Current PA (min)	Target PA (min/w)	Mon.	Tue.	Wed.	Thur.	Fri.	Sat.	Sun.
0–30	≥30	Upload last week’s completion Make plans for this week	3 days,1 time/day, 10 min/time					
30–60	≥60		4 days,1time/d					
			1 time:10 min, 2–3 time:15 min, 4 time:20 min					
60–90	≥90		4 days, 1 time/day					
			1–2 time: 20 min, 3–4 time: 25 min					
90–120	≥120		5 days, 1 time/day, 25 min/time					
120–150	≥150		5 days, 1 time/day, 30 min/time					

**TABLE 2:** List of physical activities available to participants.

01. Walking quickly	11. Badminton	21. Plank support
02. Running	12. Table tennis	22. Pushups
03. Tai Ji	13. Volleyball	23. Sit-ups
04. Skipping rope (≥100 time/min)	14. Football	24. Pull-ups
05. By bike (outdoor > 8 km/h)	15. Basketball	25. Lunge
06. By bike (gym > 8 km/h)	16. Tennis	26. Jumping jacks
07. Upstairs	17. Swimming	27. Squat
08. Downstairs	18. Martial arts	28. Pilates
09. Wash the car	19. Skating	29. Aerobic dance
10. Do housework	20. Skiing	30. Yoga

Before activity: Warm-up ≥ 5 min.

After activity: stretch ≥ 5 min.

The time of each successive activity: ≥ 10 min.

The interval between two activities: ≤ 3 days.

Please don’t be nervous if you feel strenuous or the heart rate significantly accelerated in the process of activity, this is a normal phenomenon. But if you feel unwell, please seek medical advice immediately.

#### Routine Care

All participants will receive a routine nursing care. The rehabilitation therapist will provide health education on frailty and physical activity: manifestation, risk, prevention, screening, and treatment of frailty; benefits of moderate physical activity; and introduction of common forms are listed in Table 2. A registered nurse will conduct a telephonic follow-up for all participants once in a month.

### Outcome Measures

In this study, the primary outcome will be the objective changes in physical function. At the same time, other measuring tools will be used to evaluate the multidimensional efficacy of the intervention, and a less number of participants will be selected for semi-structured interviews to gain an in-depth understanding.

#### Primary Outcome

##### Physical Function

The physical function will be evaluated by the Timed Up & Go (TUG) test (Mathias et al., 1986), which assesses the basic mobility skill as well as strength, balance, and mobility. In this test, the subjects will stand up from a standard chair (the chair with a height of 46 cm and arms with a height of 65 cm), wear comfortable shoes, walk at a regular speed of 3 m, turn around, walk back to the chair, and sit down in the chair, and then will stop the timer. According to the test results, ≤ 10 s will be considered completely independent, 10–19 s will be considered to be independent, between 20 and 29 s will be considered a “gray area,” and more than 30 s will be considered to be completely dependent. Its reliability and effectiveness have been verified in various populations (Chan et al., 2017; Yuksel et al., 2017).

#### Secondary Outcomes

##### Gait Parameters

Gait parameters include walking speed, symmetry, and variability. Participants will wear smart insoles and walk more than 25 m on barrier-free horizontal roads for at least 2 min at their self-selected comfortable pace. The walking process will include four parts, namely, acceleration, deceleration, uniform speed, and turning. The system will complete the gait analysis during this period. Walking speed (m/s) is defined as the walking distance per unit time, and when it is less than 0.6 (Zaccardi et al., 2019), there will be a higher risk of falls. Gait variability is defined as the variable coefficient of all the step-by-step cycles and is used to evaluate the variation of temporal parameters. The normal range is 2–3%, and higher values are regarded as unstable. Gait symmetry is defined as the ratio of the swinging time of biped in the air and is usually around 1.02% for healthy adults.

##### Frail Score

The frailty phenotype represents the most known operational definitions of frailty in older persons (Fried et al., 2001). According to the Fried phenotype: 0 = health; 1–2 = pre-frailty; and ≥ 3 = frailty. Walking speed, grip strength, weight, physical activity, and self-reported fatigue will be measured by the methods as shown in Table 3. One point will be recorded when any items reach the cutoff value.

**TABLE 3:** Indicators collected and assessment tools at each time point.

Test	Content				Time point				
					Screen	Baseline		12 w	24 w
FRAIL	The simple “FRAIL” questionnaire screening tool				√					
	Fatigue	Are you fatigued?							
	Resistance	Cannot walk up one flight of stairs?							
	Aerobic	Cannot walk one block (500 m)?							
	Illnesses	Do you have more than 5 illnesses?							
	Loss of weight	Have you lost more than 5% of your weight in the last 6 months?							
	Scoring: 3 or greater = frail; 1 or 2 = prefrail								
Demographic	Standardized questionnaire assessing age, gender, education level, residential status, falls history, medical history and so on.					√		√	√
Gait	Gait parameters generated by the device					√		√	√
TUG	Timed up and go test					√		√	√
Fried	Scoring: 3 or greater = frail; 1 or 2 = prefrail; 0 = health					√		√	√
	Walking speed	Slow gait: < 1.0 m/s							
	Grip strength	Measure three times and take the optimum							
		Low grip strength as follows:							

		**Males**		**Females**					
		**BMI**	**Grip strength (kg)**	**BMI**	**Grip strength (kg)**				

		≤24	≤29	≤23	≤17				
		24.1–28	≤30	23.1–26	≤17.3				
		≥28	≤32	26.1–29	≤18				
	Weight loss	Methods: measure three times and take the average, after urination in the morning							
		Weight loss: an unintentional weight loss ≥ 10 kg/10% over the previous year or an average monthly loss ≥ 1 kg/1%							
	Insufficient physical activity	≤150 min of light to moderate physical activity per week, or ≤ 75 min of vigorous physical activity per week, or the total level of physical activity did not achieve the same amount of expenditure							
	Fatigue	Asking question “Do you feel too weak to do what you want to do in the last month?” If the participants answered “yes,” they will be considered fatigued							
MVPA	Short International Physical Activity Questionnaire						√	√	√
	High	Meet any one of the following 2 standards							
		Vigorous activity ≥ 3 days and total level ≥ 1,500 MET							
		The total physical activity ≥ 7 days and 3,000 MET-min/w							
	Sufficient	Meet any one of the following 3 standards							
		Vigorous activity ≥ 20 min per day and total ≥ 3 days							
		Moderate and/or walking ≥ 30 min per day and total ≥ 5 days							
		The total physical activity ≥ 5 days and 600 MET-min/w							
	Low	Meet any one of the following 2 standards							
		No activity was reported							
		Some activities reported, but not meet the criterion of the above sufficient and high							
PHQ-9	Patients Health Questionnaire 9-item						√	√	√
GAD-7	Generalized Anxiety Disorder 7-item						√	√	√
MMSE	Mini-Mental State Examination						√	√	√
PSQI	Pittsburgh Sleep Quality Index						√	√	√

##### Moderate-to-Vigorous-Intensity Physical Activity Per Week (MET-Min/Week)

Moderate-to-vigorous-intensity physical activity (MVPA) will be evaluated by the International Physical Activity Questionnaire (IPAQ) (Bassett, 2003). The IPAQ Questionnaire comprises 7 questions about the frequency and duration of vigorous activity (8 MET), moderate activity (4 MET), walking (3.3 MET), and sitting. The total physical activity will be calculated by multiplying the time (minutes per week) by the intensity [metabolic equivalent of task (MET) unit]. Its effectiveness in evaluating the physical activity level has been verified in various populations in China (Hu et al., 2015; Ren et al., 2017). Data processing principle: the cutoff value of the daily time of a certain intensity physical activity is 180 min; if the total daily time of three intensities physical activity is > 960 min (16 h), data will be excluded from the analysis; if the daily time of a certain intensity physical activity is < 10 min, the time and corresponding weekly frequency will be recorded as 0.

##### Psychological Condition

Patients’ psychological condition will be reflected by anxiety, depression, and sleep quality. Anxiety will be evaluated by the Generalized Anxiety Disorder 7-item (GAD-7) Scale within 7 questions and a total score of 0–21 (Spitzer et al., 2006). Depression will be assessed by the Patients Health Questionnaire 9-item (PHQ-9) within 9 questions and a total score of 0–27 (Kroenke et al., 2001). The Pittsburgh Sleep Quality Index (PSQI) will be used to evaluate the sleep quality in the last month, which consists of 19 self-report items and 5 other-report items; the 19th self-report item and 5 other-report items will not be scored. The total score ranged from 0 to 21; the higher the score, the worse the sleep (Liu et al., 2021).

##### Cognitive Function

The Mini-Mental State Examination (MMSE) is one of the standardized intelligence examination tools and is widely used in the screening of Alzheimer’s disease (Folstein et al., 1975). Cognitive function will be evaluated by testing their orientation, memory, attention, calculation, recall, and language ability. The total score is related to education level with a range of 0–30.

##### Adherence and Security

If 9 weeks and more reach to the target activity levels during the entire intervention period, it will be considered as good adherence. The incidence of adverse events, including falls and all-cause hospital admissions, will be assessed by patient self-report.

##### Other Secondary Outcomes

The difference between the percentage of walking speed < 0.6m/s, gait variability > 3%, frailty score ≥ 3, and low physical activity will also be compared.

### Statistical Analysis

Categorical variables will be described by frequency and percentage. Continuous variables will be described by means and standard deviations. Social demographic and clinical data between the groups will be presented using appropriate descriptive statistics and evaluated for homogeneity using independent *t*-test, Mann-Whitney, chi-square, and Fisher’s exact tests, as appropriate. The differential changes of the primary and secondary outcomes at T1 and T2 concerning T0 between the two groups will be assessed using generalized estimating equations (GEE). The baseline variable will be adjusted, and group effects, time effects, and interaction effects will be observed. DSS statistics 25 will be used for data analysis, and *P* < 0.05 will be considered statistically significant.

### Quality Control

Our research group has established a team of experienced clinical nurses and rehabilitation therapists at a rehabilitation center in Changchun, China. Experienced clinical nurses and researchers from the team will be responsible for recruiting participants. Participants meeting the inclusion criteria will be screened, they will be informed of the study details by the investigator, and if they agree to participate, they will be asked to sign a consent form. If there is a complex clinical problem, our researchers, nursing specialists, and rehabilitation therapists will work together to find a solution.

## Discussion

The benefits of physical activity on the frail older adults have been widely recognized (Liu et al., 2018; Trombetti et al., 2018), but which part caused the improvement of health profile is still uncertain. Wearable devices have been widely used in healthcare (Chromik et al., 2022); however, it still needs to be further verified whether the remote evaluation of frailty (Angulo et al., 2020) and obtaining objective feedback can be realized. In this study, the IERMS will be used to intervene the physical activities to evaluate the changes in body performance, which will provide a new method for the remote assessment and supervision of the frail older adults living in the community.

During the COVID-19 pandemic, central-based or home-based face-to-face supervision has been forced to stop, and we need an alternative delivery mode (García Pérez de Sevilla et al., 2021). Remote assessment and guidance become the most feasible way (Liao et al., 2021). Although compared with traditional training, there is no significant difference in remote supervision, but it reached the same benefits at least (Geraedts et al., 2021; Gagnon et al., 2022). We will conduct a randomized controlled trail based on a digital platform, provide the personalized activity plan according to their own condition, and improve the compliance of schedule by increasing the interaction to ensure the intervention efficacy.

Multidimensional objective measurement reduces the subjective bias. Gait has been described as the “sixth vital sign” in recent years (Fritz and Lusardi, 2009). It is an integrative performance of body function with the complication of controlling walking and is associated with frailty (Montero-Odasso et al., 2011; Bortone et al., 2021). However, few wearable devices evaluate gait (Greene et al., 2014), due to the difficulty of obtaining gait parameters, even in today’s exponential development of digital health. Because of the lack of sensitivity and convenience, the existing methods of frail assessment are not suitable for wide-scale popularization. Through the use of smart insoles, we will be able to collect gait parameters continuously and dynamically. Gait analysis may become a new strategy to predict frailty.

However, this study still has certain limitations. First, the sample size is small and the population is limited to five communities in Changchun, so the results may not be fully promoted. Second, the training log is uploaded by the participants themselves, and there is still a certain problem with its authenticity. Finally, the body activity amount is calculated by the self-reported exercise form and metabolic equivalent, so there is a certain deviation in the calculation of exercise amount.

## Conclusion and Implications

In general, we designed a personalized remotely supervised physical activity program and expected to add effect by increasing the participation. Based on the evidence, we are convinced that this intervention program will be able to delay or reverse the progress of frailty. If the intervention produces a significant positive effect, the findings will potentially provide valuable evidence and serve as convenient and feasible strategies for primary healthcare to promote healthy aging.

## Ethics Statement

The studies involving human participants were reviewed and approved by Human Research Ethics Committee of the School of Nursing, Jilin University. The patients/participants will provide their written informed consent to participate in this study.

## Author Contributions

XZ and FL: study concept and design. XZ, JL, TZY, XL, and TYY: acquisition of data. XZ, YP, and LZ: analysis and interpretation of data. XZ, FL, and LX: drafting of the manuscript. XS, HX, and YL: critical revision of the manuscript for important intellectual content. All authors contributed to the article and approved the submitted version.

## Conflict of Interest

The authors declare that the research was conducted in the absence of any commercial or financial relationships that could be construed as a potential conflict of interest.

## Publisher’s Note

All claims expressed in this article are solely those of the authors and do not necessarily represent those of their affiliated organizations, or those of the publisher, the editors and the reviewers. Any product that may be evaluated in this article, or claim that may be made by its manufacturer, is not guaranteed or endorsed by the publisher.

## Footnotes

1
http://www.chictr.org.cn/searchproj.aspx
